# Skeletal muscle mitochondrial uncoupling in a murine cancer cachexia model

**DOI:** 10.3892/ijo.2013.1998

**Published:** 2013-06-28

**Authors:** A. Aria Tzika, Cibely Cristine Fontes-Oliveira, Alexander A. Shestov, Caterina Constantinou, Nikolaos Psychogios, Valeria Righi, Dionyssios Mintzopoulos, Silvia Busquets, Francisco J. Lopez-Soriano, Sylvain Milot, Francois Lepine, Michael N. Mindrinos, Laurence G. Rahme, Josep M. Argiles

**Affiliations:** 1 NMR Surgical Laboratory, Department of Surgery, Massachusetts General Hospital and Shriners Burn Institute, Harvard Medical School, Boston, MA 02114, USA; 2 Athinoula A. Martinos Center of Biomedical Imaging, Department of Radiology, Massachusetts General Hospital, Charlestown, MA 02114, USA; 3 Cancer Research Group, Department of Biochemistry and Molecular Biology, Faculty of Biology, University of Barcelona, 08028 Barcelona, Spain; 4 Center for Magnetic Resonance Research, Department of Radiology, University of Minnesota Medical School, Minneapolis, MN 55455, USA; 5 Molecular Surgery Laboratory, Department of Surgery, Massachusetts General Hospital and Shriners Burn Institute, Harvard Medical School, Boston, MA 02114, USA; 6 Department of Biochemistry, Stanford University School of Medicine, Stanford, CA 94305, USA; 7 INRS-Institute Armand-Frappier, University of Quebec, Laval, QC H7V 1B7, Canada

**Keywords:** skeletal muscle, cancer cachexia, mitochondria, peroxisome proliferator-activated receptor γ co-activator-1β, uncoupling protein 3, nuclear magnetic resonance spectroscopy, gas chromatography/mass spectrometry, adenosine triphosphate, tricarboxylic acid

## Abstract

Approximately half of all cancer patients present with cachexia, a condition in which disease-associated metabolic changes lead to a severe loss of skeletal muscle mass. Working toward an integrated and mechanistic view of cancer cachexia, we investigated the hypothesis that cancer promotes mitochondrial uncoupling in skeletal muscle. We subjected mice to *in vivo* phosphorous-31 nuclear magnetic resonance (^31^P NMR) spectroscopy and subjected murine skeletal muscle samples to gas chromatography/mass spectrometry (GC/MS). The mice used in both experiments were Lewis lung carcinoma models of cancer cachexia. A novel ‘fragmented mass isotopomer’ approach was used in our dynamic analysis of ^13^C mass isotopomer data. Our ^31^P NMR and GC/MS results indicated that the adenosine triphosphate (ATP) synthesis rate and tricarboxylic acid (TCA) cycle flux were reduced by 49% and 22%, respectively, in the cancer-bearing mice (p<0.008; t-test vs. controls). The ratio of ATP synthesis rate to the TCA cycle flux (an index of mitochondrial coupling) was reduced by 32% in the cancer-bearing mice (p=0.036; t-test vs. controls). Genomic analysis revealed aberrant expression levels for key regulatory genes and transmission electron microscopy (TEM) revealed ultrastructural abnormalities in the muscle fiber, consistent with the presence of abnormal, giant mitochondria. Taken together, these data suggest that mitochondrial uncoupling occurs in cancer cachexia and thus point to the mitochondria as a potential pharmaceutical target for the treatment of cachexia. These findings may prove relevant to elucidating the mechanisms underlying skeletal muscle wasting observed in other chronic diseases, as well as in aging.

## Introduction

Approximately half of all cancer patients, particularly those with cancers of the gastrointestinal tract and lung (1–3), present with cachexia, in which disease-associated metabolic changes lead to a severe loss of skeletal muscle mass (4), resulting in a body weight reduction of ≥30% (5). Given the considerable information regarding the mechanisms underlying cancer cachexia that has come to light over the past decade (1,6–33) and the absence of available curative treatments for cancer cachexia (24,27,33–61), in the present study, we examined the hypothesis of mitochondrial uncoupling in cancer cachexia. Our hypothesis is based on our previous studies on experimental models of muscle wasting (62–71).

Although several clinically relevant animal models, in which animals exhibit a cachectic state, are characterized by profound muscle wasting, there is no consensus on which model should be used for the preclinical testing of cachexia therapies (72). In this study, we used the Lewis lung carcinoma murine model (45), which was successfully used in our previous study (71). Colon 26 adenocarcinoma is also an appropriate model for examining cachexia (73–75) and murine adenocarcinoma 16 (MAC16 adenocarcinoma) is a well-established model for studies on human gastrointestinal and pancreatic cancers (76). Indeed, muscle data obtained from mice bearing MAC16 tumors (77) are in agreement with human muscle biopsy data (78) and are suggestive of mitochondrial uncoupling.

*In vivo* nuclear magnetic resonance (NMR) spectroscopy allows for the measurement of physiological biomarkers in intact cellular systems (79,80) and has been used to identify mitochondrial dysfunction in burn trauma (62), which is also characterized by severe muscle wasting. A novel ‘fragmented mass isotopomer’ approach [similar to bonded cumomer technique (81)] to the dynamic analysis of ^13^C mass isotopomer data, measured *ex vivo* by gas chromatography/mass spectrometry (GC/MS), may be used to evaluate skeletal muscle tricarboxylic acid (TCA) flux and compare flux data under different conditions. The adenosine triphosphate (ATP) synthesis rate to the TCA cycle flux ratio may then be calculated as an index of mitochondrial coupling (82,83).

Our recent NMR spectroscopy experiments, performed in conjunction with functional genomics, suggested the presence of mitochondrial dysfunction in a mouse model of cancer cachexia (71). Therefore, in the present study, we combined the same NMR technique with the GC/MS technique, to enable the assessment of the ATP synthesis rate and TCA cycle flux, respectively, and determine whether their ratio, which is an index of mitochondrial coupling, is affected in the skeletal muscle of animals in a cancer cachexia model. Our NMR spectroscopy and GC/MS experiments were complemented by genomic analysis and transmission electron microscopy (TEM) studies.

## Materials and methods

### Animals

C57Bl/6 mice (weighing 20–25 g) (Charles River Laboratories, Boston, MA, USA) were used as a representative inbred stock and reliable reference population for the microarray analyses. The animals were maintained at 22±2°C with a regular light-dark cycle (lights on from 8:00 a.m. to 8:00 p.m.) and allowed free access to standard rodent chow and water. The chow consisted of 54% carbohydrate, 17% protein and 5% fat (the remainder was non-digestible material). Food consumption was measured daily by subtracting the weight of food remaining after 24 h from the weight of food provided 24 h earlier. Daily net values of food consumption were used to determine the rate of food intake. In order to avoid the variability that may result from the female estrous cycle, only male mice were used. All animal experiments were approved by the Subcommittee on Research Animal Care of Massachusetts General Hospital, Boston, MA, USA.

### Tumor implantation

Mice were inoculated with Lewis lung carcinoma cells, according to an established protocol, under brief isoflurane anesthesia (3% in O_2_), as previously described (45, 71). Animals were randomized into tumor-free control (C) and tumor-bearing (TB) groups. Mice in the TB group were inoculated intramuscularly (right hind leg) with 4×10^5^ Lewis lung carcinoma cells obtained from exponential tumors.

### ^31^P NMR spectroscopy

NMR spectra of hind limbs were acquired 14 days after the intramuscular (hind leg) injection of 4×10^5^ Lewis lung carcinoma cells from exponential tumors. All NMR spectroscopy experiments were performed using a horizontal bore magnet (400-MHz proton frequency and 21-cm diameter; Magnex Scientific), with a Bruker Avance console. A 90° pulse was optimized for the detection of phosphorus spectra (repetition time, 2 sec; 400 averages; 4,000 data points). Saturation 90°-selective pulse trains (duration, 36.534 msec; bandwidth, 75 Hz) followed by crushing gradients were used to saturate the γ-ATP peak. The same saturation pulse train was also applied downfield of the inorganic phosphate (Pi) resonance, symmetrically to the γ-ATP resonance. T1 relaxation times of Pi and phosphocreatine (PCr) were measured using an inversion recovery pulse sequence in the presence of γ-ATP saturation. An adiabatic pulse (400 scans; sweep with 10 kHz; 4,000 data points) was used to invert Pi and PCr, with an inversion time between 152 and 7,651 msec.

### NMR spectroscopy data analysis

^31^P NMR spectra were analyzed using the MestReNova NMR software package (Mestrelab Research S.L. version 6.2.1 NMR solutions; www.mestrec.com). Free induction decays were zero-filled to 8,192 points and apodized with an exponential multiplication factor (30 Hz) prior to Fourier transformation. The spectra were then manually phased and corrected for baseline broad features with the Whittaker smoother algorithm (84). The Levenberg-Marquardt algorithm was used to least-square fit a model of mixed Gaussian/Lorentzian functions to the data. Similarly, the T1_obs_ relaxation time for Pi and PCr was calculated by fitting the function *y* = *A*1(1 − *A*2e^−(^*^t^*^/T1obs)^) to the inversion recovery data, where *y* is the *z* magnetization and *t* is the inversion time.

### Calculation of ATP concentration and synthesis rate

The ATP concentration was measured using a Bioluminescence Assay kit CLS II (cat. no. 1699695; Roche Diagnostics Corp., Indianapolis, IN, USA). Information from ^31^P NMR spectra and the previously mentioned biochemically measured concentration of ATP was used to calculate the ATP synthesis rates, as previously described by Forsen and Hoffman (85). In brief, the chemical reaction between Pi and ATP is:

[1] $$Pi\overset{k_{f}}{\to }ATP\;\;\;and\;\;\;Pi\underset{k_{r}}{\leftarrow }ATP$$

where *k_f_* and *k_r_* are the forward and reverse reaction rate constants, respectively. The influence of the chemical exchange between Pi and ATP on the longitudinal magnetization of Pi, *M_(Pi)_*, is described by:

[2] $$\frac{dM_{\left(Pi\right)}}{dt}=\frac{M_{0(Pi)}-M_{\left(Pi\right)}}{T_{1(Pi)}}-k_{f}M_{\left(Pi\right)}+k_{r}M_{\left(ATP\right)}$$

At equilibrium (*dM_(Pi)_*/*dt)* = 0 and with ATP saturation (*M*_(_*_ATP_*_)_ = 0), equation [2] becomes:

[3] $$\frac{M_{\left(Pi\right)}}{M_{0(Pi)}}=\frac{1}{1+k_{f}T_{1(Pi)}}$$

The spin lattice relaxation time, T_1app_, measured using the inversion recovery pulse sequence in the presence of ATP saturation, is related to the intrinsic T_1(Pi)_ by:

[4] $$\frac{1}{T_{1app}}=\frac{1}{T_{1(Pi)}}+k_{f}$$

Combining [3] and [4] yields:

[5] $$k_{f}=\frac{1}{T_{qpp}}\;\frac{\Delta M_{\left(Pi\right)}}{M_{0(Pi)}}$$

where *ΔM*_(_*_Pi_*_)_/*M*_0(_*_Pi_*_)_ is the fractional change of the longitudinal magnetization *M*_(_*_Pi_*_)_ of Pi. All quantities on the right side of [5] can be calculated from the NMR spectroscopy data. Finally, the unidirectional ATP synthesis flux can be calculated as:

[6] $${\left(\frac{dATP}{dt}\right)}_{synth}=k_{f}[Pi]$$

where [*Pi*] is the concentration of Pi extrapolated from a baseline NMR spectrum by comparing the peak integrals from Pi and γ-ATP, with respect to the biochemically measured concentration of ATP.

### TCA flux assessment

The TCA cycle flux was calculated from the timecourse of ^13^C mass isotopomers of glutamate (mass isotopomers M + 1 and M + 2 of glutamate), during an infusion of [2-^13^C]acetate. Plasma acetate concentration and ^13^C enrichment of glutamate in the gastrocnemius muscle were obtained by GC/MS, as previously described (86,87). A one-compartment dynamic metabolic model was used to fit ^13^C timecourses of mass isotopomers of glutamate to determine gastrocnemius metabolic flux values.

The model was mathematically expressed using 2 types of mass balance equations: i) mass balance for total metabolite concentration and ii) ^13^C mass isotopomer mass balance for labeled metabolites and their fragments, based on assumed bionetwork and atom distribution matrices (fragmented mass isotopomer framework).

In this model, the infused labeled (or unlabeled in plasma) glucose is transported from the extracellular medium to the muscle cells, assuming reversible non-steady-state Michaelis-Menten transport kinetics through the glucose transporter (GLUT4). Labeled acetate molecules are transported from the plasma to the cell interior and consequently to the mitochondria, obeying Michaelis-Menten kinetics, through the monocarboxylate transporter. The metabolic network includes glycolysis, the TCA cycle, α-ketoglutarate-glutamate and oxaloacetate-aspartate exchange, pyruvate carboxylase activity, anaplerosis at the succinyl-CoA level, pyruvate recycling through malic enzyme and acetyl-CoA synthetase activity. Once acetyl-CoA is formed, either through the pyruvate dehydrogenase complex, β-oxidation or acetyl-CoA synthetase, it is used in the TCA cycle to produce energy and electron carriers.

Mass isotopomer balance equations were derived in a similar manner as the equations for bonded cumulative isotopomers (e.g., for glutamate, glutamine and aspartate), as previously described by Shestov *et al*(81). This study resulted in a set of 75 non-linear mass isotopomer ordinary differential equations. The concept of fragmented mass isotopomers, as well as bonded cumulative isotopomers (81), or *bonded cumomers*, leads to a reduced number of equations, as well as a more simple derivation of equations, compared to a model that includes all possible isotopomers, while retaining all mass spectrometry-measurable mass isotopomer information.

In terms of ordinary differential equations, the model describes the rates of loss and formation of particular labeled and unlabeled metabolite forms (mass isotopomers), following infusion of a labeled substrate. These equations are based on the flux balance of metabolites and take the form (e.g., for parallel unimolecular reactions):

$$[M]\frac{d\mu_{\left(i\right)}}{dt}=\sum_{j}F_{j}\sigma_{j(i)}-\left(\sum_{k}F_{k}\right)•\mu_{\left(i\right)}$$

where metabolite M is downstream of another metabolite, S_j_. The total outflux $\sum_{k}F_{k}$ balances the total influx ∑*F*_j_. [M] represents the total pool size of metabolite M, while μ_(i)_ and σ_j(i)_ represent the I mass isotopomer fraction of metabolite M (M + I mass isotopomer) and metabolite S_j_ (S + I mass isotopomer). The number of labeled C atoms in molecules, I, changes between 0 and N, where N is the total number of C atoms in the metabolites.

For labeled [2-^13^C]acetate infusion, the fitted timecourses were Glu M + 1 and Glu M + 2 mass-isotopomers, with a total of two curves. The two following fluxes were determined: gastrocnemius TCA cycle F_TCA_ and exchange flux between glutamate and 2-oxoglutarate F_X_. Solving a system of non-linear differential equations in terms of whole/fragmented mass isotopomers, with the Runge-Kutta 4th order procedure for stiff systems, yields timecourses for all possible ^13^C mass isotopomers (e.g., glutamate, glutamine and aspartate). The cost function is used to quantify differences between measurements and computational results for labeled dynamic data. Minimization was performed with Broyden-Fletcher-Goldfarb-Shanno (BFGS) or Simplex algorithms. Proper mean-square convergence was confirmed by verifying that goodness-of-fit values were close to the expected theoretical values. We estimated errors for the obtained values using Monte Carlo simulations with experimental noise levels. All numerical procedures were carried out using Matlab software.

### Transmission electron microscopy

For mitochondrial morphology analysis, gastrocnemius muscles were extracted, dissected into small sections (~1 mm^2^) and transferred to glass vials filled with 2% paraformaldehyde and 2.5% glutaraldehyde in cacodylate buffer (fixative buffer). The samples were maintained in the fixative buffer for 24 h at 4°C. The samples were rinsed 4 times with the same buffer (10 min per rinse) and post-fixed in 1% osmium tetroxide in the same buffer containing 0.8% potassium ferricyanide for 90 min at 4°C. The dehydration procedure was performed in different dilutions of acetone in distilled water (50, 70, 90, 96 and 100% v/v). The dehydrated samples were infiltrated with Epon resin over 2 days, embedded in the same resin and allowed to polymerize at 60°C for 48 h.

Resin-embedded specimens were cut into semi-thin sections using an ultramicrotome (Reichert-Jung Ultracut E) and the sections were stained with bromophenol blue, to visualize cell borders under a light microscope (Leica). When skeletal muscle fibers were detected and selected, we proceeded to collect ultra-thin sections using a Leica Ultracut UCT ultramicrotome and mounted the ultra-thin sections on Formvar-coated copper grids. The mounted sections were stained with 2% uranyl acetate in water and lead citrate. The stained sections were observed under a JEM-1010 electron microscope (Jeol, Tokyo, Japan) with an acceleration voltage of 80 kV. Images were captured using a CCD camera (MegaView III) and digitized by software-supported analysis (Soft Imaging System, Münster, Germany).

### RNA extraction, gene array hybridization and data analysis

Muscle specimens were homogenized in phosphate-buffered saline (PBS) for 60 sec using a tissue homogenizer (Brinkman Polytron PT3000), followed by TRIzol RNA extraction (Gibco-BRL). Total RNA was purified using an RNeasy kit (Qiagen, Germantown, MD, USA). RNA purity and quantity were assessed by spectrophotometry and capillary gel electrophoresis (Agilent 2100) and the RNA samples were stored at −80°C. Biotinylated cRNA was extracted from 10-mg samples of total RNA and hybridized to GeneChip^®^ Mouse Gene 1.0 ST arrays, which were then stained, washed and scanned. All procedures followed standard Affymetrix, Inc. protocols (Santa Clara, CA, USA) and all experiments were performed in triplicate and analyzed as described in our previous publications (62,63).

## Results

**Figure 1:**
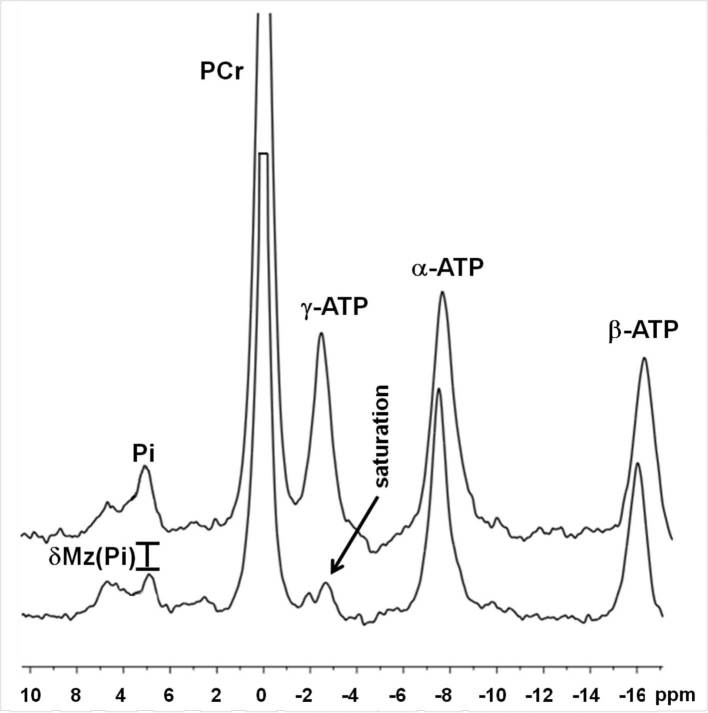
NMR spectra from *in vivo*^31^P NMR spectroscopy saturation-transfer experiments performed on the hind limb skeletal muscle of control (C) mice for determination of unidirectional inorganic phosphate (Pi) to ATP flux. Representative summed ^31^P-NMR spectra acquired from C before (upper curve) and after (lower curve) saturation of the γ-ATP resonance. The arrow on γ-ATP indicates the position of saturation by radiofrequency irradiation (−13.2 ppm). ppm, chemical shift in parts per million.

Fig. 1 shows representative ^31^P NMR spectra acquired from the mice in the C group before and after saturation of the γ-ATP resonance. Upon irradiation of the γ-ATP resonance, the signal intensities for the PCr, Pi, α-ATP and β-ATP resonances were all decreased, either by magnetization transfer or by direct off-resonance saturation.

We discovered that the unidirectional synthesis rate of the Pi → γ-ATP reaction in the mice in the TB group was 49% lower compared to that observed in the mice in the C group (p=0.008). Additionally, the unidirectional synthesis rate of the PCr → γ-ATP reaction was 22% lower in the mice in the TB group compared to those in the C group, a difference that was significant according to a unidirectional (one-tailed) t-test (p=0.036). The NMR-measured fractional change in magnetization (Δ*M*/*M_0_*) was decreased by 37% in the TB group compared to the C group. The ATP concentration (14 days post-inoculation) was lower in the mice in the TB group compared to those in the C group by approximately 27%, a difference that approached significance (p=0.054) in the unidirectional (one-tailed) t-test.

**Figure 2:**
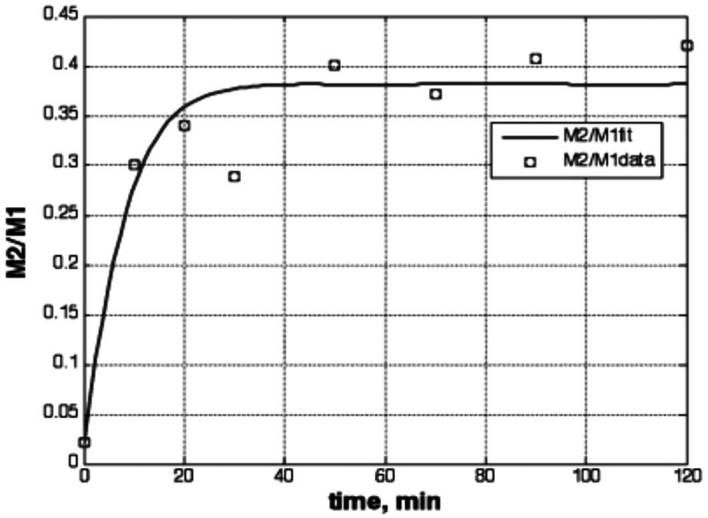
Dynamic profile for M + 2 to M + 1 ratio (M2/M1) of glutamate mass isotopomers during [2-^13^C]acetate infusion in mouse exhibiting cancer cachexia. Squares indicate experimental data points, and the line represents the best fit for our dynamic mass isotopomer model.

**Figure 3:**
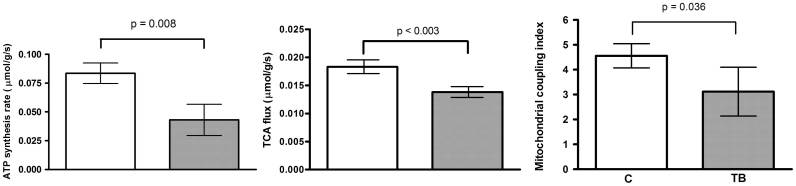
Rates of unidirectional ATP synthesis (left plot), TCA cycle flux (TCA, middle plot) and the coupling index (right plot), calculated as the ratio of the former two variables in control (C) and tumor-bearing (TB) mice.

The experimental timecourse for the labeled glutamate mass isotopomer ratio M + 2/M +1 obtained during the infusion of [2-^13^C]acetate is presented in Fig. 2. As shown in Table I, we found that the ATP synthesis rate, calculated from ^31^P NMR spectroscopy data, and the TCA cycle flux, calculated from mass spectrometry data, were reduced by 49 and 25%, respectively, in the mice in the TB group (p=0.008 and p<0.003; Mann-Whitney U test). The ratio of the ATP synthesis rate to the TCA cycle flux (an index of mitochondrial coupling) was 32% lower in the mice in the TB group compared to those in the C group (p=0.036; Mann-Whitney U test; Table I). Fig. 3 graphically depicts the results presented in Table I.

**Figure 4:**
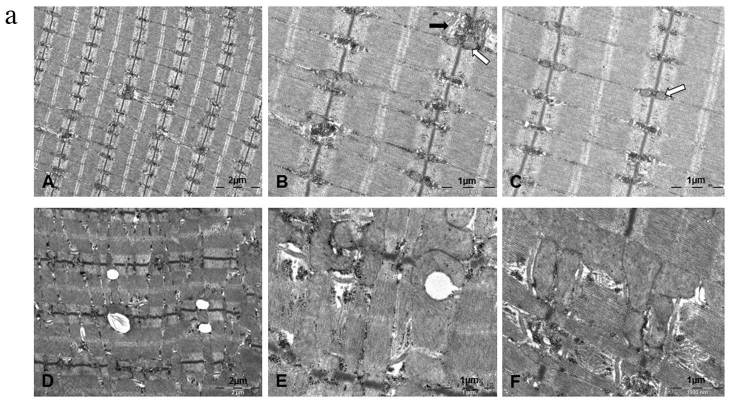

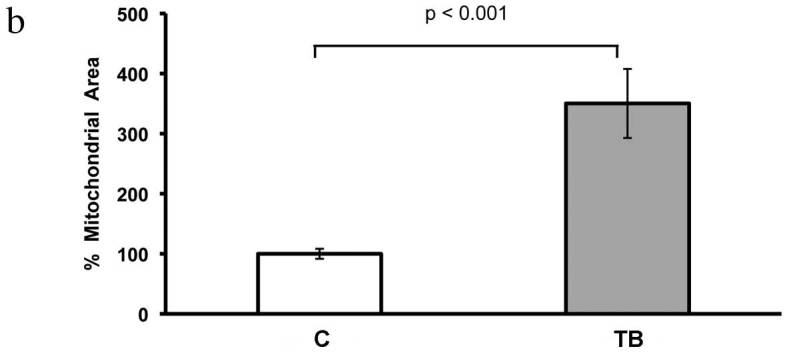
(a) TEM micrographs from healthy control muscle (A–C) and cancer-induced cachectic (D–F) gastrocnemius muscle. White arrows, normal mitochondria; black arrow, triad structure. (b) Barograph showing the difference in the mitochondrial area between control and cancer-induced cachectic gastrocnemius muscle.

Images from our TEM observations showing features of gastrocnemius muscle morphology are presented in Fig. 4a. In gastrocnemius muscle samples from mice in the C group, we observed normal myofibrils with a clear division of bands and an intact sarcomere structure (Fig. 4a-A). Mitochondria in the control specimens were distributed along the Z line (Fig. 4a-B and -C, white arrows) and adjacent to triad structures (Fig. 4a-B, black arrow). In mice in the TB group, gastrocnemius muscle micrographs from the leg contralateral to the cancer cell inoculation were characterized by disorganization of myofibrils (Fig. 1D), giant mitochondria and lipid accumulation (Fig. 4a-E and -F). Moreover, the intermyofibrillar mitochondria area was increased by 3.5-fold (p<0.001) in cachectic gastrocnemius muscle from mice in the TB group (Fig. 4b).

Gene expression analysis demonstrated that, compared to mice in the C group, mice in the TB group exhibited an upregulated expression of uncoupling protein 3 (UCP3; p=0.03), forkhead box O 3α (FOXO3α; p=0.04) and atrogin-1 (p=0.0084), as well as an upregulated expression of pyruvate dehydrogenase kinase 4 (PDK4; p=0.01), an inhibitor of the pyruvate dehydrogenase complex. The expression of peroxisome proliferator-activated receptor γ coactivator-1β (PGC-1β) was significantly downregulated in the mice in the TB group (p=0.04 vs. C group). These results corroborate, in part, previous findings (19,71,88).

## Discussion

The principle finding of this study was that TCA cycle flux determined by mass spectrometry was significantly reduced in mice in the TB group, compared to those in the C group. We further found that the ATP synthesis rate, determined by ^31^P NMR spectroscopy, was significantly reduced in mice in the TB group, which was consistent with our previous findings and suggestive of bioenergetic mitochondrial dysfunction (71). The ATP synthesis rate to the TCA cycle flux ratio (an index of mitochondrial coupling) was also reduced in mice in the TB group. In addition, our TEM observations revealed disrupted muscle morphology in mice in the TB group and our gene expression experiments demonstrated an upregulation of UCP3, FOXO3α, atrogin-1 and PDK4 expression, accompanied by a downregulation of PGC-1β expression. The novel results reported in this study provide evidence of mitochondrial uncoupling in cancer cachexia and demonstrate that cancer-induced cachexia leads to a profound functional and structural disorganization of murine skeletal muscle. Our data corroborate, in part, previous findings obtained from other experimental models (19,71,77,88–90), as well as data from human muscle biopsies (78).

To elucidate the function of gastrocnemius TCA flux in cancer cachexia, we used ^13^C-labeling experiments. We employed a novel fragmented mass isotopomer approach for the dynamic analysis of ^13^C mass isotopomer data measured *ex vivo* by GC/MS. Our dynamic labeling approach has several advantages compared to the steady-state isotopic approach commonly used for such studies. The main advantage of our approach is that it provides absolute flux values instead of flux ratios at network branch points. Secondly, it provides quantitative understanding of metabolic networks and enables researchers to discriminate between multiple pathways that give similar labeled metabolite patterns in steady-state analyses. Thirdly, our dynamic analysis approach requires less labeling time and thus requires less labeling compounds, which are costly. Finally, dynamic models enable researchers to gain a better insight into metabolic networks.

Of note, our NMR spectroscopy findings of mitochondrial dysfunction in the skeletal muscle of animals exhibiting cancer cachexia, are similar to observations previously made on murine models of burn trauma (62,68). *In vivo*^31^P NMR spectroscopy saturation transfer may be used to measure fast enzyme reaction exchange rates non-invasively (91) and, in particular, the net rate of oxidative ATP synthesis catalyzed by mitochondrial ATPase in skeletal muscle, which is proportional to oxygen consumption (92,93). It has been proposed that NMR-measured unidirectional ATP synthesis flux primarily reflects flux through F1F0-ATP synthase, with negligible influence of the coupled glyceraldehyde-3-phosphate dehydrogenase and phosphoglycerate kinase reactions (82,94). Since these enzymes are present at near-equilibrium levels, the unidirectional production of ATP may be pronounced. Furthermore, since PDK4 expression is upregulated in cancer cachexia in mice, as shown in this study (Fig. 3) and in our previous study (71), as well as in rats (95), we hypothesized that the contribution of glycolytic reactions to unidirectional ATP synthesis flux is negligible, since PDK4 inhibits the pyruvate dehydrogenase complex, which is involved in controlling the use of glucose-linked substrates as sources of oxidative energy via glycolysis (96).

The aberrant expression of genes involved in mitochondrial biogenesis (PGC-1β) and uncoupling (UCP3) that we observed in this clinically relevant cancer cachexia model, are in agreement with previously reported data (71). The reduced expression of PGC-1β has also been observed in murine models of burn-induced skeletal muscle wasting (62,68), whereas increased PGC-1α protein levels have been reported in a rat cancer cachexia model (95), strongly suggesting that PGC-1 proteins play a key role in cancer-induced muscle wasting. Specifically, it has been suggested that PGC-1α protects skeletal muscle from atrophy (25), while PGC-1β expression has been associated with an increase in ATP-consuming reactions (97). Moreover, reduced PGC-1 expression levels have also been correlated with profoundly reduced mitochondrial content and activity (98). These effects may be due to the action of UCPs (65), given that PGC-1 downregulation is accompanied by increased UCP expression in murine models of both cancer- (89,90,95) and burn-related (62,65) cachexia. Increased levels of UCPs dissipate the proton gradient and lower the mitochondrial membrane potential, a process that increases energy expenditure by dissipating energy as heat (99). The presently observed upregulation of UCP3 in cancer cachectic animals agrees with our mitochondrial coupling index difference in suggesting the presence of mitochondrial uncoupling in cachectic mice and corroborates previous findings (78,88–90).

Our results are consistent with earlier studies on rats, in which an increase in UCP3 expression, induced by T3 treatment (94) or fasting (82), was associated with an increase in mitochondrial uncoupling. However, in contrast to these earlier studies, wherein the observed increase in mitochondrial uncoupling was the result of an increased TCA cycle flux (i.e., the denominator), the change in coupling that we observed in our cancer-bearing mice was the result of changes in both the ATP synthesis rate (the numerator) and TCA cycle flux (the denominator). Since the decrease in ATP synthesis rates was more pronounced than in the TCA cycle flux, the ratio (coupling index) was decreased. Our data are further supported by a previous report, demonstrating significantly increased mitochondrial coupling in UCP3 knockout mice (87).

Our TEM findings demonstrated that cancer-induced cachexia causes a profound structural disorganization of skeletal muscle characterized by fiber disruption, band disarrangement and dilated sarcoplasmic reticulum. In addition, the intermyofibrillar area in specimens from cachectic mice was characterized by the presence of giant mitochondria, which are formed when dysfunctional mitochondria are unable to achieve fusion (100). Swollen mitochondria have previously been described in cancer-induced cachexia (101). These giant mitochondria point to a causal effect of structural mitochondrial dysfunction on cancer-induced muscle wasting. In light of our previously published genomic data (71), the presently observed increase in intramyocellular lipids in specimens from mice in the TB group may be attributed to defective intracellular lipid metabolism. It has also been suggested that increased intramyocellular lipids may signal apoptosis (63), a process known to contribute to cancer-induced muscle wasting that was induced in our experimental murine model of cancer-induced cachexia (102).

In conclusion, our study fills a knowledge gap in an integrated and mechanistic view of cancer cachexia. Since the muscle wasting that occurs in cancer is similar to that observed in a number of other chronic diseases and aging, studies that focus on the mechanisms underlying cachexia are of significant public health relevance. Muscle biopsies from cachectic patients with pancreatic (103) and gastrointestinal cancer (26,78) have been reported to show supra-normal mitochondrial uncoupling (78), apoptosis and DNA fragmentation (26), as well as evidence of oxidative stress resulting from reactive oxygen species (ROS), a fact corroborated by animal studies (71,88,104). Although the integrity of mitochondrial DNA (mtDNA) was not examined in this study, such effects may be inferred, given that mtDNA is highly vulnerable to oxidative damage induced by ROS, as it is situated closer to the site of ROS generation, lacks protective histones and has more limited base excision repair mechanisms than nuclear DNA (66,105–111). Further studies on the effects of cancer cachexia on mtDNA integrity, as well as studies designed to test mitochondrial agents are warranted.

## Figures and Tables

**Table I:** ATP synthesis, TCA cycle flux and mitochondrial coupling index mean values (± standard errors) in control and tumor-bearing mice.

Variable	C	TB	% Change	p-value
ATP synthesis rate (μmol/g/sec)	0.084±0.009 n=10	0.043±0.013 n=6	−48.8	0.008
TCA flux (μmol/g/sec)	0.018±0.005 n=18	0.014±0.003 n=18	−22.2	<0.003
Mitochondrial coupling index^a^	4.559±0.486	3.117±0.979	−31.6	0.036

Mann-Whitney U test was used for the comparisons.

a Calculated per animal as the ratio of ATP synthesis rate to the TCA flux.

C, control mice; TB, tumor-bearing mice; n, number of mice.
